# Divergent color signals from homologous unfeathered ornaments in two congeneric grouse

**DOI:** 10.1002/ece3.5687

**Published:** 2019-09-27

**Authors:** Geoffrey M. Gould, Gerald G. Carter, Jacqueline K. Augustine

**Affiliations:** ^1^ Department of Evolution, Ecology and Organismal Biology The Ohio State University Columbus OH USA; ^2^ Department of Evolution, Ecology and Organismal Biology The Ohio State University at Lima Lima OH USA

**Keywords:** character displacement, honest signaling, lek‐mating grouse, signal divergence, skin color, spectrometry

## Abstract

Color‐based visual signals are important aspects of communication throughout the animal kingdom. Individuals evaluate color to obtain information about age and condition and to behave accordingly. Birds display a variety of striking, conspicuous colors and make ideal subjects for the study of color signaling. While most studies of avian color focus on plumage, bare unfeathered body parts also display a wide range of color signals. Mate choice and intrasexual competitive interactions are easily observed in lekking grouse, which also signal with prominent unfeathered color patches. Most male grouse have one pair of colorful bare part ornaments (combs), and males of several species also have inflatable air sacs in their throat. Previous studies have mostly focused on comb color and size, but little is known about the signaling role of air sac color. We measured comb size and the color properties of combs and air sacs in the Lesser and Greater Prairie‐Chickens (*Tympanuchus pallidicinctus* and *T. cupido*, respectively), and investigated whether these properties varied with age and mass. We found that mass predicted color properties of air sacs and that age predicted comb size in the Greater Prairie‐Chicken, suggesting that these ornaments indicate condition dependence. No conclusive relationships between color and age or size were detected in the Lesser Prairie‐Chicken. Color properties of both ornaments differed between the two species. Further research is needed to determine mechanisms that link condition to color and whether the information advertised by color signals from these ornaments is intended for males, females, or both.

## Graphical abstract

We measured comb size and the color properties of combs and air sacs in wild caught Lesser and Greater Prairie‐Chickens (*Tympanuchus pallidicinctus* and *T. cupido*, respectively), and investigated whether these properties varied with age and mass. We found that mass predicted color properties of air sacs and that age predicted comb size in the Greater Prairie‐Chicken, but no conclusive relationships between ornament color and age or size were detected in the Lesser Prairie‐Chicken. Our findings suggest the occurrence of a past episode of character displacement, possibly as a means of discouraging hybridization, between these recently diverged congeneric species.

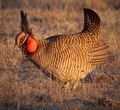

## 1 INTRODUCTION

Color signals play an important role in visual signaling and communication throughout the animal kingdom. The wide variety of bright and conspicuous colors observed in birds make avian systems ideal for studying the signaling functions of color. There is abundant evidence for overall coloration and color patches acting as signals in important behavioral interactions such as mate choice (Hill, 2006), intrasexual competition (Senar, 2006), and parental provisioning (Kilner, 2006). Color signals convey information regarding the physical state of individuals such as growth rate (Doucet & Montgomerie, 2003), parasite loads (Mougeot, Redpath, & Leckie, 2005), immune response (Griggio, Hoi, & Pilastro, 2010; Jourdie, Moureau, Bennett, & Heeb, 2004; Peters, Denk, Delhey, & Kempenears, 2004), and testosterone levels (Peters, Delhey, Goymann, & Kempenears, 2006; Roberts, Ras, & Peters, 2009). Color expression predicts outcomes with critical fitness implications, such as probability of survival (Sheldon, Andersson, Griffith, Örnborg, & Sendecka, 1999), latency to establish a territory (Siefferman & Hill, 2005; Siitari & Huhta, 2002), likelihood of experiencing cuckoldry (Delhey, Johnsen, Peters, Andersson, & Kempenaers, 2003), and ability to secure extrapair mating (Freeman‐Gallant et al., 2010; Sirkiä & Laaksonen, 2010).

Models of honest advertisement predict that overall coloration and ornamental size and color can act as reliable signals of male condition. These models are most often applied to sexual selection and lead to the hypothesis that females prefer to mate with maximally ornamented males in order to procure “good genes” for their offspring (Hamilton & Zuk, 1982; Kodric‐Brown & Brown, 1984; Zahavi, 1975). Honest advertisement models also apply to signals used in male–male competition, as signal interpretation can influence decisions to escalate agonistic encounters or adjust display intensity (Grafen, 1990). Theoretical support for honest advertisement models is based on the assumption that the expression of ornaments is condition‐dependent (Borgia, 1979, Pomiankowski & Møller, 1995, Rowe & Houle, 1996, reviewed in Kotiaho & Puurtinen, 2007). As expected, abundant empirical evidence has shown that color signals reliably advertise condition in a variety of avian families (Dobson et al., 2008; Doucet, 2002; Griggio, Hoi, & Pilastro, 2010; Keyser & Hill, 1999; Zirpoli, Black, & Gabriel, 2013).

Color can also convey age, and the perception of age influences many social decisions in birds and other taxa. Females prefer to mate with older males (Beck & Powell, 2000; Manning, 1985), so the evaluation and comparison of color‐based signals can therefore allow females to identify males that are older and therefore preferred (Brooks & Kemp, 2001; Kokko, 1988; Proulx, Day, & Rowe, 2002). Older male birds often display larger or more colorful patches to advertise their dominance (Rohwer, 1977), while younger birds may benefit from honest advertisement of their youth through color signaling by experiencing reduced aggression from more experienced rivals (Senar, 2006).

Although the signaling function of avian color has been widely studied, most published studies have focused on the signaling characteristics of plumage (Hawkins, Hill, & Mercadante, 2012; Hill, 2006; LaFountain, Prum, & Frank, 2015; McGraw, 2006); the role of color arising from bare parts such as bills, legs, and skin has received considerably less attention (Iverson & Karubian, 2017). The Lesser Prairie‐Chicken (*Tympanuchus pallidicinctus*) and Greater Prairie‐Chicken (*T. cupido*) are promiscuous grouse which copulate and engage in aggressive intrasexual interactions on aggregated leks. Males of these species exhibit two pairs of brightly colored fleshy ornaments which are featured prominently in mating displays—supraorbital combs and esophageal inflatable apteria (hereafter “air sacs”). These color ornaments contrast strongly against their otherwise cryptic plumage which helps to camouflage prairie‐chickens in their grassland habitat. It is therefore likely that these color patches fulfill an important signaling role. Copulation events and intrasexual dominance encounters are easily observed on aggregated leks, and thus, these species are well suited for studying the relationship between color expression, individual characteristics, and behavior.

Age predicts mating success in lekking grouse (Alatalo, Höglund, Lundberg, & Sutherland, 1992) including the Lesser Prairie‐Chicken (Behney, Grisham, Boal, Whitlaw, & Haukos, 2012), possibly because yearlings may lower their reproductive effort and subsequently benefit from higher survival (Hagen, Pitman, Sandercock, Robel, & Applegate, 2005). Comb size and condition predict mating success in several grouse species (Hannon & Eason, 1995; Holder & Montgomerie, 1993; Rintamäki et al., 2000) including the Greater Prairie‐Chicken (Augustine, Millspaugh, & Sandercock, 2011; Nooker & Sandercock, 2008). The size of grouse combs also correlates with hormonal status (Moss et al., 1979; Stokkan, 1979a), decreased endoparasite loads (Vergara, Mougeot, Martíınez‐Padilla, Leckie, & Redpath, 2012), mating success (Hannon & Wingfield, 1990), social rank (Gjesdal, 1977; Holder & Montgomerie, 1993; Myhre, 1980; Stokkan, 1979b), and the ability to hold territories (MacColl, Piertney, Moss, & Lambin, 2000). Fewer studies have examined possible effects of comb color properties, and most of these focused on the relationship between color and parasites (Mougeot, Redpath, & Leckie, 2005, Martínez‐Padilla, Mougeot, Pérez‐ Rodríguez, & Bortolotti, 2007, Martínez‐Padilla, Mougeot, Webster, Pérez‐ Rodríguez, & Piertney, 2010, Mougeot, Martínez‐Padilla, Bortolotti, Webster, & Piertney, 2010, but see Yang, Wang, Fang, & Sun, 2013, Harris, 2016).

The role of the visual properties of air sacs in intraspecific communication has only been examined in the Greater Sage‐Grouse (*Centrocercus urophasianus*). In this species, females avoided males whose air sacs had hematomas which were possibly caused by louse infestation (Johnson & Boyce, 1990), and females also avoided males with artificially applied hematomas in laboratory mate choice trials (Spurrier, Boyce, & Manly, 1991). The three species in the *Tympanuchus* genus display esophageal air sacs, but there has been no study of the relationship between the color properties of these fleshy structures and age or physical characteristics such as body mass or size in these species. More generally, the color signaling functions of bare parts in birds are much less studied relative to plumage‐based color signaling, and this study attempts to address this gap through quantitative assessment of bare part color signals.

Here, we investigated whether comb size, comb color, and air sac color are predicted by age or mass in the Lesser and Greater Prairie‐Chickens. We tested if color signals correlate with mass and age differentially between the two species. Because age and condition are expected to be assessed by conspecifics in both intra‐ and intersexual contexts, we expected age and condition to predict the color properties of prominently displayed ornaments. If so, these ornaments may act as reliable signals for evaluation. Due to the importance of age in grouse mating systems, we predicted that older males would have larger combs and that their ornaments would be brighter and have increased saturation. We also expected that males in better condition (i.e., heavier) would have greater ability to allocate resources to ornamental structures and therefore we predict that heavier males would have larger, brighter, and more color‐saturated combs. Finally, we expected that comb characteristics would vary with age and size in both species.

## 2 METHODS

### 2.1 Study species

The Lesser and Greater Prairie‐Chickens are obligate grassland grouse (Subfamily Tetraonidae). Males of both species perform mating displays on aggregated leks during the breeding season (Wiley, 1974). Stereotyped displays are characterized by rapid foot stomping, extension of pinnae feathers above the head, and postures featuring tail fanning and wing spreading. Intrasexual agonistic interactions occur in both sexes although they are more common and more intense in males (Hjorth, 1970; Sharpe, 1968). Males of both species have two pairs of unfeathered secondary ornaments. Supraorbital combs are raised as a result of increased blood flow to the tissues (Hollett, Thomas, & MacDonald, 1984) and are usually visible when males are in attendance at leks. Air sacs are inflated to produce vocalizations through the contraction of specialized muscles in a broadened portion of the esophagus (Potapov & Sale, 2013).

**Figure 1:**
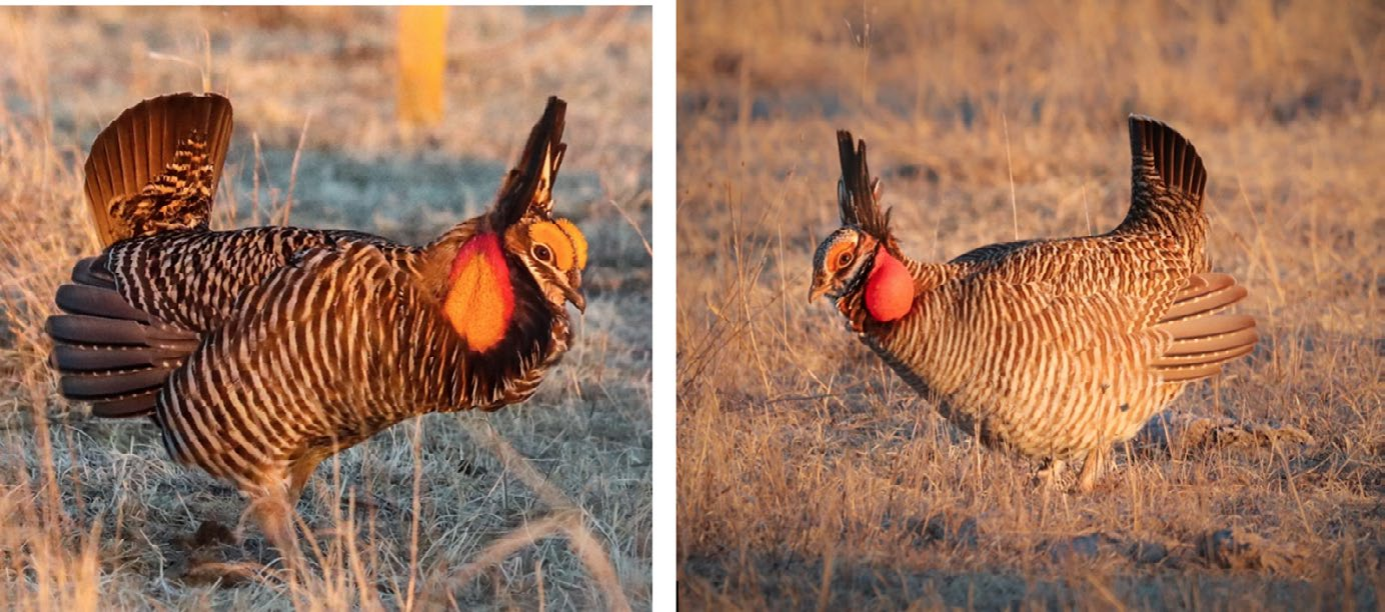
Color ornaments above the eye (comb) and in the throat (air sac) in the Greater (left) and Lesser (right) Prairie‐ChickensGeoffrey M. Gould took the photos

There are several differences in appearance between the two species. Greater Prairie‐Chickens are larger and have darker plumage overall, and bars on belly feathers are wider and extend further toward the tail (Short, 1967). Greater Prairie‐Chickens have larger air sacs which appear orange, while the smaller air sacs of Lesser Prairie‐Chickens have a reddish appearance (Jones, 1964; Sharpe, 1968; Figure 1). Display vocalizations produced by inflation of the air sacs are easily distinguished with Greater Prairie‐Chickens sounding long low frequency notes, while Lesser Prairie‐Chickens produce higher frequency short bursts often referred to as “gobbling” (Jones, 1964).

### 2.2 Field methods

We measured comb and air sac color of live male Lesser (*N* = 100) and Greater Prairie‐Chickens (*N* = 24) captured on leks during the breeding season in the spring of 2012–2013 and 2016–2018. Males were trapped opportunistically using mechanical drop nets on leks in Trego, Gove, and Riley Counties in Kansas, USA. Each bird was aged as a yearling (first breeding season, Lesser Prairie‐Chicken *N* = 41; Greater Prairie‐Chicken *N* = 8) or an adult (subsequent breeding seasons, Lesser Prairie‐Chicken *N* = 59, Greater Prairie‐Chicken *N* = 16) using the shape and coloration of the outermost wing feather (Copelin, 1963). We recorded the mass of each bird (±1 g) with a digital scale (Ohaus) and used digital calipers to measure the length and height of combs (±0.01 mm).

We used a portable JAZ Ocean Optics spectrometer with a pulsed xenon light source to obtain reflectance spectra spanning the visible and UV portions of the electromagnetic spectrum (300–700 nanometers), which corresponds to the UV sensitivity of birds in the order Galliformes (Bowmaker, Heath, Wilkie, & Hunt, 1997; Hart, Partridge, & Cuthill, 1999; Wortel, Rugenbrink, & Nuboer, 1987). The probe was connected to the processing unit with a fiber optic cable and was mounted within a holder to ensure that all readings were taken at a 45 degree angle, 10 mm from the skin being measured. All measurements were taken relative to a >98% white reflectance standard (PTFE optical diffuser). We obtained at least three spectra from 1 mm diameter areas from one comb and air sac for each male, repositioning the probe for each reading resulting in a total of at least six readings for each individual. All experimental procedures were conducted under the approval and guidance of The Ohio State University's Institutional Animal Care and Use Committee (IACUC Protocol # 2011A00000023 and 2013A00000013) and under permits issued by the Kansas Department of Wildlife, Parks and Tourism (Permit # SC‐016‐2012, SC‐029‐2013, SC‐048‐2016, SC‐038‐2017, and SC‐060‐2018).

### 2.3 Quantification of color within avian (tetrahedral) color space

**Figure 2:**
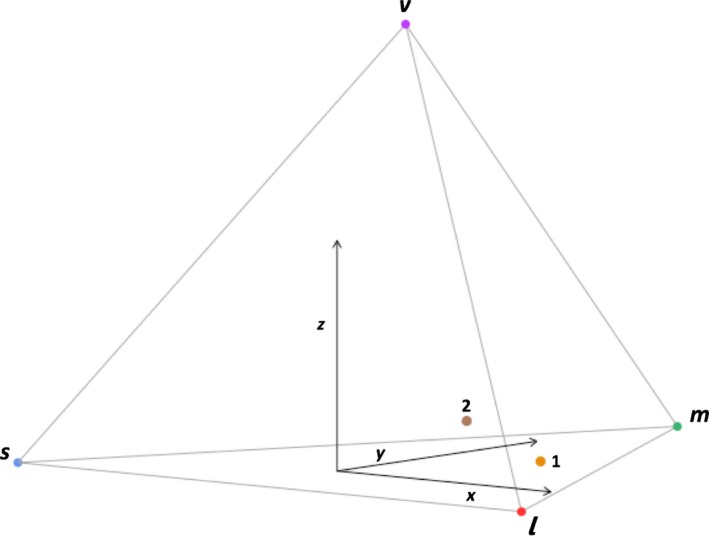
Average spectra of all air sacs from Greater Prairie‐Chickens (Point 1) and Lesser Prairie‐Chickens (Point 2) plotted in tetrahedral color space. The vertex labels correspond to the four types of retinal cones in galliform species (s = short, m = medium, and l = long wave, and v = violet). The origin is offset for visual clarity

Although color stimuli are often quantified based on the shape and position of spectra obtained from color patches (colorimetric variables), color stimuli can also be described using visual modeling techniques which quantify the stimuli as they would be perceived by the visual system of the focal species. Analyses of this type are preferable for studies which aim to link behavioral responses to color stimuli, as they provide a better approximation of how the receivers perceive these stimuli. Burkhardt (1989) and Goldsmith (1990) described a tetrahedral color space for the avian visual system in which any source of light, such as a spectrum or average of several spectra, can be plotted as a point within a tetrahedron (Figure 2). Each corner of the tetrahedron corresponds to one of the four types of color receptors (ultraviolet/violet, short wave, medium wave, and long wave), and the distance from the point representing the light source to each vertex of the tetrahedron is based on the expected absorption of each of the four types of receptors, which is a function of wavelength (Vorobyev & Osoroio, 1998). In addition, color perception is affected by factors extrinsic to the stimulus and visual system such as ambient light conditions (Endler & Mielke, 2005).

**Figure 3:**
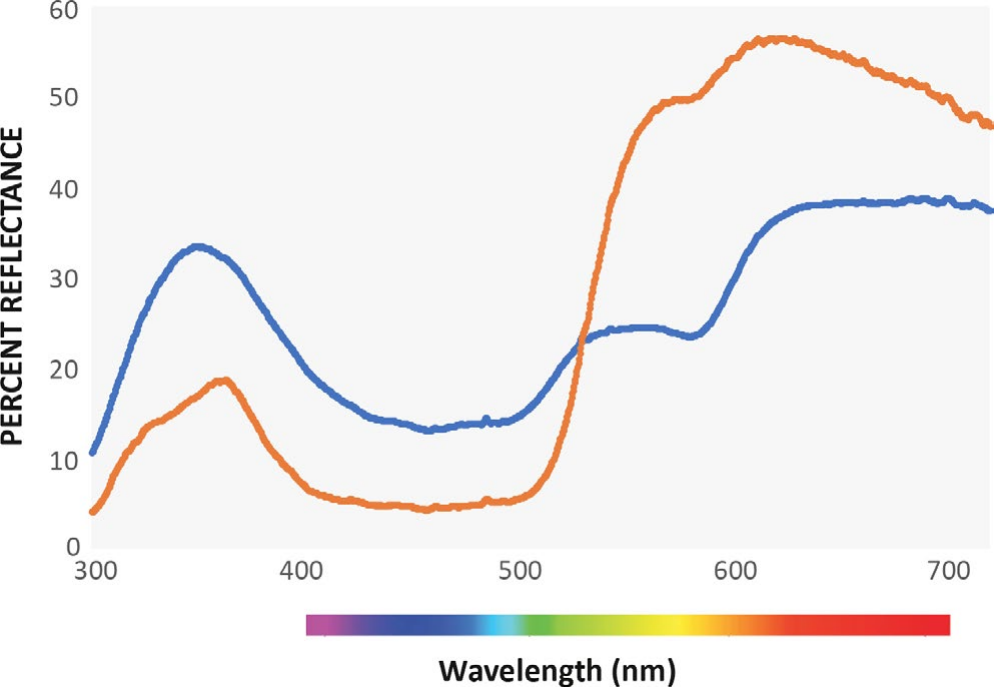
Average of all Lesser Prairie‐Chicken (blue, *N* = 380) and Greater Prairie‐Chicken (orange line, *N* = 96) spectra taken from air sacs used in this study

**Figure 4:**
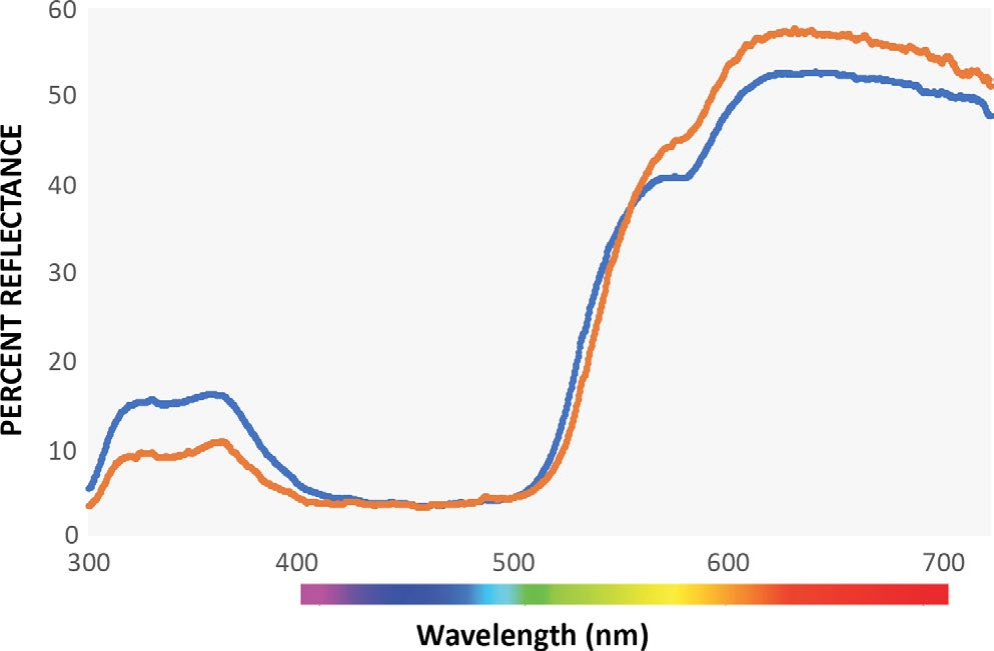
Average of all Lesser Prairie‐Chicken (blue, *N* = 377) and Greater Prairie‐Chicken (orange, *N* = 96) spectra taken from combs used in this study

The *pavo* package in R (Maia, Eliason, Bitton, Doucet, & Shawkey, 2013) provides a computational framework which allows for the extraction of traditional color variables such as hue, brightness, and saturation from input full spectrum data, in addition to modeling color within a variety of color spaces such as the avian tetrahedral color space. In our analysis of the relationship between color and condition or age, we chose variables based on the tetrahedral color space to account for bimodal spectra such as those arising from prairie‐chicken ornaments (Figures 3 and 4). The three chromatic variables we used describe the length and angle of the vector that connects the point representing the color stimulus in tetrahedral space to the origin of the tetrahedron. Hue is described by two variables: φ (UV hue) describes the vertical angle of the vector relative to the plane formed by the *X*‐ and *Y*‐axes of the tetrahedron, and θ (non‐UV or RGB hue) describes the horizontal angle of the vector relative to the positive *X*‐axis (spectra with higher θ values are located further from the *X*‐axis in the direction of the line connecting the medium and short wave vertices in Figure 2). Chroma or saturation is described by r‐achieved which is the ratio of the length of the vector to its maximum possible length for the given hue (Stoddard & Prum, 2008). Brightness (described by the “luminance” variable in *pavo*) is the achromatic component of a color signal and is a function of the intensity of a color signal's reflectance over the entire range of wavelengths under consideration (300–700 nm in our analysis). We obtained four measurements (UV hue(φ), non‐UV or RGB hue(θ), chroma or saturation (r‐achieved), and brightness (luminance)) for comb and air sac separately to obtain eight color variables for each individual bird.

While the precise spectral sensitivity of retinal cones has not been empirically determined for prairie‐chickens, avian color visual systems are often grouped into two broad categories: the passerine and galliform eye (Cuthill et al., 2000). As an approximation for the prairie‐chicken visual system, we therefore used the relative cone abundances for the peafowl (*Pavo cristatus*) and domestic chicken (*Gallus gallus domesticus*) provided by the *pavo* package for chromatic and achromatic visual perception properties, respectively. We used the D65 setting to model the background light environment as standard daylight.

Due to the imbalance in sample sizes between the two species in our data set, we compared color variability of both ornaments for the Lesser and Greater Prairie‐Chickens in *pavo*. Among the summary statistics available after situating multiple spectra in tetrahedral color space is the volume of the minimum convex polygon that contains all input spectra, with greater volume indicating greater variability.

### 2.4 Visual models for color distinguishability

In order for a stimulus to act as an intraspecific signal, there must be variation in its expression and this variation must be distinguishable in the perceptual system of the species. To test if color expression in the Lesser and Greater Prairie‐Chickens is distinguishable to conspecifics, we used the coldist function within *pavo* to calculate color distances with the receptor noise visual model of Vorobyev and Osoroio (1998) which is based on the relative densities of the different types of retinal cones that process color stimuli. The distances generated by this function correspond to distances between points in tetrahedral space. To test distinguishability under avian visual models, we performed a series of pairwise contrasts of the chromatic and achromatic features of full spectra for individuals exhibiting the highest and lowest values of saturation and brightness for both of our study species. Contrasts are reported as ΔS for chromatic differences and ΔL for achromatic differences using units of just‐noticeable differences (JND). Values >1.0 JND indicate distinguishability under the modeled visual system with values >2.0 representing easily distinguishable contrasts (Jones & Siefferman, 2014; Jones et al., 2017).

### 2.5 Statistical analysis

We used body mass as our explanatory variable to test the relationship between condition (body mass) and the color expression and size of combs and air sacs. Body condition indices such as ratio indices (Sijbranda, Campbell, Gartrell, & Howe, 2016; Ware, McClure, Carlisle, & Barber, 2015), residuals of mass‐structural measure regressions (Dobson et al., 2008; McGraw, Massaro, Rivers, & Mattern, 2009; Mougeot & Arroyo, 2006; Schulte‐Hostedde, Zinner, Millar, & Hickling, 2005), and scaled mass indices (David, Auclair, Dall, & Cézilly, 2013; Galbraith, Stanley, Jones, & Beggs, 2017; Peig & Green, 2009) are often used as proxies for overall condition, but the relationship of these indices to protein and lipid profiles varies between and among species and populations. Therefore, in the absence of validation experiments relating proxy condition indices to fitness‐relevant physical characteristics, body mass alone can serve as a reliable proxy for condition (Labocha & Hayes, 2012; McGuire et al., 2018).

We used linear regressions to examine the relationship between comb area and comb color variables and between comb area and body mass. Comb area was estimated as comb length * height (Mougeot, Martínez‐Padilla, Pérez‐Rodriguez, & Bortolotti, 2007). For each ornament, we averaged the spectra generated from all color readings. To investigate whether mass or age predicted the expression of color variables, we used linear mixed models (LMM) with six fixed effects: species, mass, age, the interaction between mass and age, the interaction between mass and species, and the interaction between age and species. Year and lek of capture were random effects. Response variables were comb size or the expression of four color variables each for the comb or the air sac, leading to a total of nine LMM's. To account for multiple models, we applied the sequential Bonferroni–Holm method (Wright, 1992). A *p*‐value for each model was generated by comparing the full model to an intercept‐only model (without fixed effects), and we considered terms to retain significance only if the full model *p*‐value remained below .05 after applying the Bonferroni–Holm correction. To test each term, we used likelihood ratio tests of the full model with the effect in question against the model without the effect in question. To interpret a significant interaction between species and another effect, we plotted the bootstrapped 95% confidence intervals for the standardized model coefficients for each species (*boot* package in R, 1,000 iterations). Residual plots did not reveal any obvious deviations from homoscedasticity or normality. Analyses were performed in R (R Core Team, 2016) using the *lme4* package.

## 3 RESULTS

### 3.1 Color comparison of Lesser and Greater Prairie‐Chickens

Lesser and Greater Prairie‐Chickens differed in the color of their bare part ornaments. The air sacs differed by saturation, luminance, and UV hue (Table 1). The combs differed by UV hue and RGB hue (Table 1).

**Table 1:** Results of linear mixed models describing variation in comb and air sac color variables and comb area in relation to age, mass, species, and interactions of age*species and mass*species in Lesser (*N* = 100) and Greater (*N* = 24) Prairie‐Chickens

Ornament	Variable	Predictor	Coefficient ± *SE*	*T*	*p*	Corrected full model *p*‐value
Comb	θ (RGB hue)	Mass	0.2924 ± 0.31	0.93	.35	**.01**
		Age	1.77 ± 1.41	1.26	.21	
		Species	1.62 ± 0.65	2.504	**.014**	
		Mass*Species	−0.24 ± 0.37	−0.62	.53	
		Age*Species	−1.77 ± 1.43	−1.24	.22	
	φ (UV hue)	Mass	−0.32 ± 0.35	−0.93	.35	.13
		Age	−1.57 ± 1.59	−0.99	.32	
		Species	−1.61 ± 0.73	−2.322	**.028**	
		Mass*Species	−0.04 ± 0.42	−0.10	.92	
		Age*Species	1.82 ± 1.61	1.13	.26	
	R‐achieved (saturation)	Mass	−0.41 ± 0.76	1.24	.22	.58
		Age	−0.30 ± 1.68	−0.18	.86	
		Species	−0.96 ± 0.76	−1.27	.21	
		Mass*Species	−0.07 ± 0.44	−0.17	.87	
		Age*Species	0.51 ± 1.70	0.30	.77	
	Luminance	Mass	−0.05 ± 0.37	−0.14	.89	.58
		Age	−0.42 ± 1.65	−0.26	.80	
		Species	−0.18 ± 0.75	−0.24	.81	
		Mass*Species	0.54 ± 0.44	1.24	.22	
		Age*Species	0.21 ± 1.66	0.13	.90	
	Comb area	Mass	−0.79 ± 0.32	−2.44	**.02**	.14
		Age	−3.14 ± 1.44	−2.18	**.03**	
		Species	−1.15 ± 0.67	−1.72	.09	
		Mass*Species	0.75 ± 0.39	−1.92	.06	
		Age*Species	2.99 ± 1.47	−2.04	**.04**	
Air sac	θ (RGB hue)	Mass	0.78 ± 0.31	2.54	**.0125**	**<.0001**
		Age	1.02 ± 1.39	0.74	.46	
		Species	−0.27 ± 0.63	−0.42	.68	
		Mass*Species	−0.97 ± 0.37	−2.65	**.0091**	
		Age*Species	−1.15 ± 1.42	−0.81	.42	
	φ (UV hue)	Mass	−0.72 ± 0.31	−2.36	**.02**	**.0041**
		Age	−1.60 ± 1.38	−1.16	.25	
		Species	−2.29 ± 0.63	−3.64	**.0004**	
		Mass*Species	0.96 ± 0.36	2.628	**.0097**	
		Age*Species	1.93 ± 1.40	1.38	.17	
	R‐achieved (saturation)	Mass	−0.51 ± 0.19	−2.64	**.0095**	**<.0001**
		Age	−1.00 ± 0.86	−1.17	.24	
		Species	−2.91 ± 0.40	−7.34	**<.0001**	
		Mass*Species	0.47 ± 0.23	2.03	**.0451**	
		Age*Species	1.08 ± 0.87	1.24	.22	
	Luminance	Mass	0.11 ± 0.24	0.47	.64	**<.0001**
		Age	−1.76 ± 1.05	−1.67	.10	
		Species	−1.31 ± 0.48	−2.72	**.0077**	
		Mass*Species	−0.19 ± 0.28	−0.69	.49	
		Age*Species	1.70 ± 1.07	1.59	.11	

Note

A separate analysis was performed for each color variable, and year and lek of capture were included as random factors. *p*‐values <.05 shown in bold for individual terms and full models corrected for multiple comparisons. The reference categories for age and species are yearling and Lesser Prairie‐Chicken, respectively.

**Figure 5:**
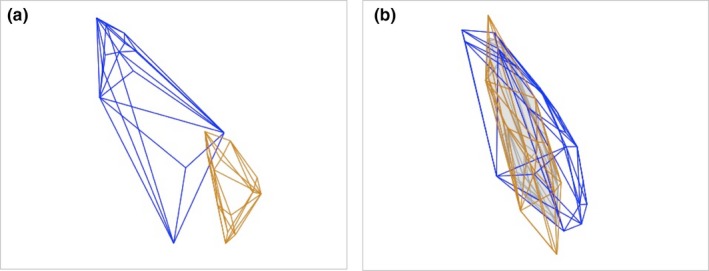
Representation of color volume in tetrahedral space for Lesser (blue polygons) and Greater (orange polygons) Prairie‐Chickens for air sacs (Panel a) and combs (Panel b). The shaded area represents the area of overlap for the combs

The Lesser Prairie‐Chicken had a larger color volume (variability) for both ornaments despite having a larger sample size within our dataset (air sac: Lesser Prairie‐Chicken volume = 2.84 × 10^−4^, Greater Prairie‐Chicken volume = 2.81 × 10^−5^, Figure 5a; comb: Lesser Prairie‐Chicken volume = 8.26 × 10^−5^, Greater Prairie‐Chicken volume = 2.03 × 10^−5^, Figure 5b).

We calculated the coefficient of variation (standard deviation/mean) for the achromatic portion of the color stimuli (brightness). For air sac brightness, the coefficient of variation was 32.04% for Lesser Prairie‐Chickens and 30.77% for Greater Prairie‐Chickens. For comb brightness, the coefficient of variation was 25.10% for Lesser Prairie‐Chickens and 32.51% for Greater Prairie‐Chickens.

### 3.2 Effect of age and mass on ornament color

**Figure 6:**
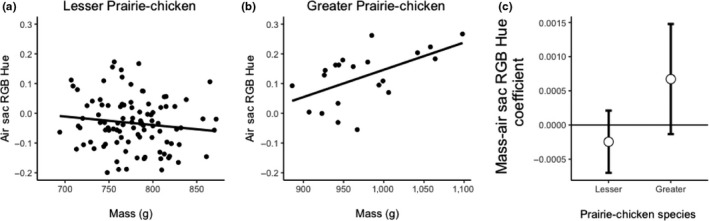
Comparison of mass and air sac non‐UV (RGB) hue (θ) in Lesser and Greater Prairie‐Chickens. (a) Air sac RGB hue does not correlate with mass in Lesser Prairie‐Chickens. (b) Air sacs of heavier Greater Prairie‐Chickens have higher RGB hue values. (c) Bootstrapped 95% confidence intervals of the standardized slope estimates for the effect of mass on air sac RGB hue using 1,000 permutations of the linear mixed model

**Figure 7:**
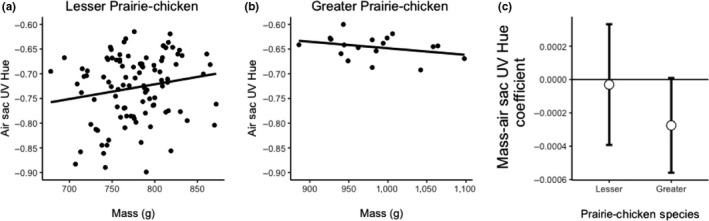
Comparison of mass and air sac UV hue (φ) in Lesser and Greater Prairie‐Chickens. (a) Air sac UV hue does not correlate with mass in Lesser Prairie‐Chickens. (b) Air sac RGB hue approaches significance, but does not correlate with mass in Greater Prairie‐Chickens. (c) Bootstrapped 95% confidence intervals of the standardized slope estimates for the effect of mass on air sac UV hue using 1,000 permutations of the linear mixed model

**Figure 8:**
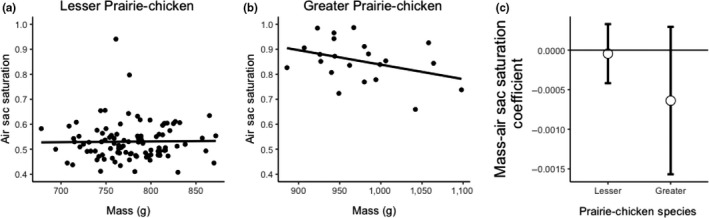
Comparison of mass and air sac saturation (r‐achieved) in Lesser and Greater Prairie‐Chickens. (a) Air sac saturation does not correlate with mass in Lesser Prairie‐Chickens. (b) Air sac saturation does not correlate with mass in Greater Prairie‐Chickens. (c) Bootstrapped 95% confidence intervals of the standardized slope estimates for the effect of mass on air sac saturation using 1,000 permutations of the linear mixed model

We found that the relationship between mass and RGB hue (θ) differed by species (Table 1), with post hoc tests showing that mass predicted air sac RGB hue in Greater Prairie‐Chickens, but not Lesser Prairie‐Chickens. We did not detect an interaction between mass and air sac RGB hue (θ) in the Lesser Prairie‐Chicken (*R* ^2^ < .01, *p* = .46, *F* = 0.57, *N* = 100; Figure 6a), but the effect was evident in the Greater Prairie‐Chicken and air sac RGB hue increased with mass (*R* ^2^ = .27, *p* = .013, *F* = 7.46, *N* = 24; Figure 6b). The 95% confidence intervals of the effects for the two species overlapped each other, and they overlapped zero, but Greater Prairie‐Chickens generally had a positive coefficient, whereas Lesser Prairie‐Chickens had a negative coefficient (Figure 6c). We also detected interactions between mass and species for air sac UV hue (φ) and air sac saturation (Table 1). Again, post hoc linear regressions of these variables on mass showed no relationship between color and mass in Lesser Prairie‐Chickens (UV hue: *R* ^2^ = .02 *p* = .12, *F* = 2.47, Figure 7a; saturation: *R* ^2^ < .01, *p* = .86, *F* = 0.03, Figure 8a), but a weak negative trend for the Greater Prairie‐Chicken (UV Hue: *R* ^2^ = .16, *p* = .06, *F* = 3.90, Figure 7b; saturation: *R* ^2^ = .14, *p* = .09, *F* = 3.13, Figure 8b). The relationships between the two species for the bootstrapped 95% confidence intervals for UV hue and saturation were similar in that the coefficients for Greater Prairie‐Chickens were negative and showed less overlap with zero than for Lesser Prairie‐Chickens (Figures 7c and 8c).

**Figure 9:**
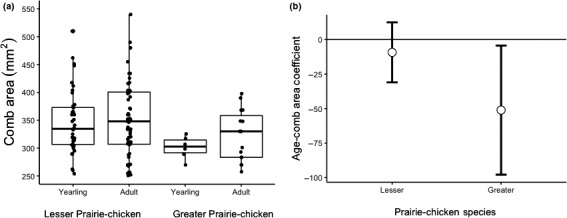
Comparison of comb area and age in Greater and Lesser Prairie‐Chickens. (a) Older Greater Prairie‐Chickens have larger combs than yearlings; Lesser Prairie‐Chicken comb size does not vary with age. (b) Bootstrapped 95% confidence intervals of the standardized slope estimates for the effect of age on comb area using 1,000 permutations of the linear mixed model

We also found evidence that the effect of age on comb area differed by species (Table 1); however, this interaction term did not retain significance when accounting for multiple comparisons. A post hoc *t* test comparing adult to yearling Greater Prairie‐Chickens showed that adult birds might have larger combs ( |t| = 2.18, *p* = .041; Figure 9a) while we detected no difference in comb area between adult and yearling Lesser Prairie‐Chickens (|*t*| = 0.27, *p* = .79; Figure 9a). Again, the 95% confidence intervals of the effects for the two species overlapped, and the effect was not detected in the Lesser Prairie‐Chicken (Figure 9b).

### 3.3 Correlations between ornament color and comb size

We did not detect correlations between comb size and any color variables for the Lesser or Greater Prairie‐Chicken. For Lesser Prairie‐Chickens (*N* = 99), comb size did not differ with mass (*F* = 1.79, *p* = .18) or comb color (UV hue *F* = 0.83, *p* = .36; RGB hue *F* = 0.20, *p* = .65; saturation *F* = 0.26, *p* = .61; luminance *F* = 0.00, *p* = 1.00). For Greater Prairie‐Chickens (N= 24), comb size did not differ with mass (*F* = 0.28, *p* = .60) or comb color (UV hue *F* = 0.03, *p* = .86; RGB hue *F* = 0.88, *p* = .36; saturation *F* = 0.01, *p* = .91; luminance *F* = 0.53, *p* = .48).

### 3.4 Visual modeling of color distinguishability

Full spectra comparison of individuals with the highest and lowest values of saturation showed that ornament color is highly distinguishable in chromatic variation for both species (Lesser Prairie‐Chicken: air sac ∆S = 18.59 JND, comb ∆S = 18.58 JND; Greater Prairie‐Chicken air sac ∆S = 20.08 JND, comb ∆S = 17.67 JND). Air sacs and combs for individuals with the most extreme values for brightness for both species were shown to be highly distinguishable in achromatic properties (Lesser Prairie‐Chicken: air sac ∆L = 18.93 JND, comb ∆L = 3.97 JND; Greater Prairie‐Chicken: air sac ∆L = 15.14 JND, comb ∆L = 9.00 JND).

## 4 DISCUSSION

We examined whether age and body mass predict the color properties and size of bare part ornaments in the Lesser and Greater Prairie‐Chickens and if color signals convey different information between the two species. The results of our models of avian vision showed that differences between individuals are distinguishable under the visual system of both species of prairie‐chickens. We found evidence that mass predicts air sac color characteristics and age predicts comb area in the Greater Prairie‐Chicken with heavier birds having higher RGB hue (θ) values, lower UV hue (φ) values, and lower saturation values and older birds having larger combs. We could not detect similar relationships in the Lesser Prairie‐Chicken.

In three cases (RGB hue, UV hue, and saturation), we found evidence for an effect of mass on air sac color in only one species, but we lacked the statistical power to conclusively show that the effects were different or the same between the two species. Our results were consistent with several possible scenarios. First, an effect might exist in only one species. Second, there could be effects in both species that differ in effect size, and we only detected the larger effect. Third, there might be the same effect in both species, and we lacked the power to detect it in one of the species. Fourth, there could be no effect in either species and we detected a false positive in one species.

In the species comparison of age and comb area, we suspect that our results are best explained by the second scenario (a larger effect in one species). While the confidence interval for the effect in the Lesser Prairie‐Chicken does contain zero, it is biased in the same direction as for the Greater Prairie‐Chicken (Figure 9b). The conspicuous appearance of combs during the lekking behavior of the Lesser Prairie‐Chicken, the ubiquitous presence of combs in grouse worldwide, and the large body of evidence supporting a signaling role for combs (Harris, 2016; Martínez‐Padilla, Mougeot, Pérez‐Rodríguez, & Bortolotti, 2007 Martínez‐Padilla, Mougeot, Webster, Pérez‐Rodríguez, & Piertney, 2010; Moss et al., 1979; Mougeot et al., 2010, 2005; Stokkan, 1979a; Vergara et al., 2012; Yang et al., 2013) suggest that the comb likely fulfills a signaling function in the Lesser Prairie‐Chicken albeit to a lesser degree than in the Greater Prairie‐Chicken.

In the case of mass and air sac UV hue (φ) and saturation, we believe that our results are best explained by the first scenario (an effect in only one species). We base this interpretation on the effect size, and the clear species differences in size, color (Figure 1), and sound produced by the air sacs, which support the interpretation of the air sac acting as a divergent signal. Additionally, because air sacs are displayed much less frequently than combs, they are less likely to serve as visual signals in all of the grouse species which display them.

In the case of mass and air sac RGB hue (θ), an effect may have existed in only one species or opposite effects might exist in each species and we lacked the statistical power to detect both of them (Figure 6). The lack of a strong correlation between air sac RGB hue and mass (*R* ^2^ < .01) in the Lesser Prairie‐Chicken provides evidence against air sac color fulfilling a signaling function in this species.

One potential explanation for the presence of bright coloration in tissues not used as visual signals relates to the biological functions of carotenoid compounds, which likely contribute to the colorful appearance of air sacs in prairie‐chickens. Both carotenoids themselves and vitamin A, for which carotenoids serve as precursors (Simpson, 1983), have been linked with wound healing properties in the epidermal tissue of a variety of vertebrates (Meephasnan, Rungjang, Yingmema, Deenonpoe, & Ponnikorn, 2017; Polcz & Barbul, 2019). Although it is difficult to determine if prairie‐chickens specifically target combs and air sacs during aggressive encounters, we have observed injuries to combs and air sacs.

The finding that air sac color indicates the condition of one of our focal species suggests that air sacs may have a signaling function independent of sound production. To our knowledge, the color characteristics of air sacs in male grouse have not been shown to be related to any aspect of health or age in previous studies. In addition to our two focal species, male Sharp‐tailed Grouse (*T. phasianellus*) and Blue Grouse (*Dendragapus* spp.) have esophageal air sacs while Sage‐Grouse (*Centrocercus* spp.) have pectoral air sacs. In all of these species, these brightly colored patches of bare skin are inflated frequently during breeding displays, both in the presence and absence of females. While the role of air sac appearance in intrasexual competition has not been examined, there is evidence that females visually inspect air sacs and that visual properties of these ornaments influence mate choice (Johnson & Boyce, 1990; Spurrier et al., 1991). Our results suggest the potential for visual signaling functions of these structures and that relationships between air sac coloration and individual characteristics such as health, age, and condition merit further study.

Body mass is a common target of sexual selection, and mass often determines the outcome of male–male agonistic dominance encounters in avian species including grouse (Kervinen, Lebigre, & Soulsbury, 2016; Nooker & Sandercock, 2008; Rintamäki, Höglund, Alatalo, & Lundberg, 2001), and conspecific behavioral decisions are likely influenced by mass due to its connection with condition. Our finding that condition was signaled by RGB hue was unexpected, as brightness and saturation represent the quality of a color, whereas hue represents the color's shade. However, there is abundant evidence that hue in the visible portion of the spectrum reflects aspects of individual quality such as age (Marini, McKellar, Ratcliffe, Marra, & Reudnik, 2015), parasite loads (Brawner, Hill, & Sundermann, 2000), response to immune challenge (Nolan, Dobson, Dresp, & Jouventin, 2006), white blood cell levels (Figuerola, Muñoz, Gutiérrez, & Ferrer, 1999), and levels of environmental toxins (García‐Heras et al., 2017). The studies cited here used methods other than modeling within tetrahedral color space such as digital photography analysis and colorimetric variables to determine hue. Whether a higher or lower hue value indicates the quality of an individual depends on the species in question. With colorimetric variables, a higher hue value corresponds to a shift toward red, but the results of color space models do not correspond directly to perceptual experience as hue variables correspond to the stimulation of photoreceptors as opposed to the direct visual experience of a color's shade (Stoddard & Stevens, 2011). Regardless of how the signal may be perceived in terms of actual color perception, our findings provide evidence that higher hue values in the air sac may indicate better condition in the Greater Prairie‐Chicken.

Bare part ornaments may be especially reliable indicators of an individual's current condition because they can respond rapidly to environmental changes (Biard, Hardy, Motreuil, & Moreau, 2009; Sternalski et al., 2010; Vergara, Fargallo, & Martíınez‐Padilla, 2015). Previous studies have shown that carotenoid levels and color properties of bare part ornaments change in response to manipulations of testosterone (Blas, Pérez‐Rodríguez, Bortolotti, Vinuela, & Marchant, 2006) and parasite loads (Martínez‐Padilla et al., 2007, 2010). Mass is expected to fluctuate over the course of a breeding season due to the energetically demanding nature of breeding displays on grouse leks (Lebigre, Alatalo, & Siitari, 2013; Siitari, Alatlalo, Halme, Buchanan, & Kilpimaa, 2007; Vehrencamp, Bradbury, & Gibson, 1989); however, individuals in our study area are typically captured once each season so the degree to which mass fluctuates remains unknown. Differences in capture date could affect color expression due to the energetic demands of breeding displays, and capture date may therefore act as a confounding factor. However, we did not detect any strong correlations between capture date and color variables or comb area in either species (linear regression, all *R* ^2^ < .19). Despite the ability of bare part ornaments to respond rapidly to changing conditions, we are not aware of any evidence for changes occurring on the scale of minutes or due to changes in blood flow. As the average processing time for an individual is about 15 min, we do not expect that changes in color expression would result from the capture process. Experimental manipulations relating to color expression have not been performed on our focal species; therefore, the other qualities that may affect color, besides mass, have not been tested and are a potential area for further study.

Further research is needed to examine the relationship between color and behavior in our focal population. Conspicuous color patches play a prominent role in mediating intrasexual male encounters in birds (Senar, 2006), and color patches can act as signals of resource holding potential which is used to assess opponents in agonistic interactions (Balzarini, Tasborsky, Villa, & Frommen, 2016; Dawkins & Guilford, 1993; O'Connor, Metcalfe, & Taylor, 1999; Sabol, Hellmann, Gray, & Hamilton, 2017; Xu & Fincke, 2015). Male prairie‐chickens frequently engage in these types of interactions during which combs and air sacs are displayed to conspecifics at close range. Color may be correlated with features of behavior which in turn may be affected by physiological factors such as the action of testosterone or parasite loads. There is abundant evidence for a link between comb size and testosterone in grouse, and comb size is often described as an androgen‐dependent character (Martínez‐Padilla et al., 2010; Pérez‐Rodriguez, Martínez‐Padilla, & Mougeot, 2013; Vergara et al., 2012). As a result, larger combs are often correlated with enhanced copulatory success, perhaps due to the influence of testosterone on increased display rates, success in intrasexual competition, or both (Augustine, Millspaugh, & Sandercock, 2011). Given our results linking comb size to age, it is possible that age, hormone levels, and ornament size interact in the context of intraspecific signaling although we acknowledge that the relationship between age and comb size we found was weak and did not retain statistical significance after accounting for multiple comparisons.

Air sacs are displayed in both a relaxed and inflated state, and it is therefore possible that they act as different signals when in these differing states, as the appearance of hues spanning a wide range of the spectrum can change as a result of stretching of biological tissues (Kolle et al., 2013; Teyssier, Saenko, Marel, & Milinkovitch, 2015). We recorded color on relaxed air sacs as they are held in this state for a longer duration and because recording color from relaxed air sacs of live birds is safer and more easily performed in the field. To artificially inflate air sacs for color readings would require forcing air into the trachea, a procedure which comes with a high risk of injury to the bird, and we felt it was necessary to minimize the risk of harming our focal species especially given that they are both species of conservation concern (BirdLife International, 2016, 2018). Thus, while inflated air sacs may act as a different signal from relaxed air sacs, we predict that variation between individuals in the appearance of inflated air sacs will correlate to the variation in relaxed air sacs.

Color characteristics may be important during species recognition given that they differ between the Lesser and Greater Prairie‐Chickens for both combs and air sacs. Due to changing land‐use practices, Lesser and Greater Prairie‐Chickens have recently begun to occupy a zone of sympatry (Van Pelt et al., 2013). Putative hybrid individuals have been observed in western Kansas (Bain & Farley, 2002), and introgression is occurring between the two species (Oyler‐McCance et al., 2016). Although putative hybrid male prairie‐chickens have been observed displaying on leks, there have been no confirmed reports of these individuals copulating successfully. If hybrid males do not reproduce, interspecific mating would be maladaptive and secondary ornaments would be expected to undergo character displacement (Lemmon & Lemmon, 2010; Pfennig & Pfennig, 2010; Ritchie, 2007; Weissing, Edelaar, & Doorn, 2011). Further study incorporating color measurements of putative hybrids may inform our understanding of the potential adaptive consequences of hybridization. Our finding that comb and air sac color differ in our two focal species and that the information signaled by ornaments might also differ between them is consistent with an episode of past character displacement in these closely related species. A similar trajectory of phenotypic displacement is theorized to have occurred with differences in display behavior in recently diverged populations of Sage‐Grouse (*Centrocercus* spp., Young, Hupp, Bradbury, & Braun, 1994).

In conclusion, our data show that air sac coloration is predicted by mass and that comb size is predicted by age in the Greater Prairie‐Chicken and that ornaments encode different information about the signaling individual between our two recently diverged congeneric focal species. Capturing individuals multiple times in a season can allow us to determine how color signals and mass fluctuate over time and if conspecific receivers adjust their behavior in relation to these fluctuations. Future research can determine if these color signals are intended to be received by males, females, or both, and can also help to establish the causal link between condition and color which would allow for these signals to be described in terms of honest advertisement models including good genes models of sexual selection.

## CONFLICT OF INTEREST

None declared.

## AUTHOR CONTRIBUTIONS

GMG and JKA designed and executed the study. GGC contributed to the statistical analysis. All authors contributed substantially to the manuscript.

## Data Availability

All data used in this manuscript have been uploaded to Dryad. Data available from the Dryad Digital Repository: https://doi.org/10.5061/dryad.ms33h5h
